# Maternity Insurance

**Published:** 1911-05-27

**Authors:** 


### MATERNITY INSURANCE.

### WHAT THE GOVERNMENT BILL WILL DO FOR WOMEN.

While doubtless the Government Bill for Compulsory Insurance will have to undergo many criticisms, it is obvious that, so far as the sickness insurance is concerned, there will be accorded to it a cordial welcome. One of the proposals which has met with the most universal favour is that providing for the inevitable expenses of childbirth. The allowance of thirty shillings to women to cover the needs of the four weeks, including, and subsequent to, the birth, may not seem a great deal, but it is an enormous advance on the existing state of things, and means that many women will have proper care and aid in childbirth and necessary rest afterwards. That this will react most beneficially on the health of both mother and child cannot be doubted, and Mr. Lloyd George is to be congratulated on having had the courage and wisdom to make the payment of the subsidy contingent on the mother not returning to her work until the four weeks are over. There will be evasions of the law—as of what law are there not! But the convention of four weeks at home once established, and the obviously greater vigour of the mother who takes full benefit of her enforced holiday proved, the idea of a sufficient period of repose after childbearing will take root among the poor, and may even in time affect those mothers who do not go out to work, and who consequently take up their usual occupations—the daily scrubbing and the weekly day at the wash-tub, not to speak of the cooking and mending, far too soon. Seven and sixpence a week may seem a small sum for the expenses of such a time, but as that is the average wage of the woman worker, it may be taken as quite reasonable. Those who earn more may be able to make some extra provision privately, while it will be a great boon to those who earn less.

### Maternity Insurance Abroad.

Perhaps it is rather humiliating to think that in the matter of maternity insurance Britain is only following the lead of nations which we do not regard as being so far advanced as ourselves. Thus, in Spain, where however a woman is allowed to return to her work three weeks after the birth of her child, the employer is compelled to pay her wages during her absence. In Austria medical aid is given, and a daily subsidy equal to 60 per cent. of the earnings. In Germany there is a compulsory sickness insurance, which gives aid for four weeks after confinement. The woman may return to work after four weeks if she produces a medical certificate of good enough health; failing that she is not permitted to go back to work until six weeks have elapsed, but in this case the law advises aid for the further period. Moreover, under the German insurance laws it is now possible to insure for sick pay up to the full amount of the earnings, and this benefit may continue for twelve weeks after confinement. In Switzerland an enforced abstention from work of eight weeks is enjoined, of which six weeks must be after the birth; but there seems to be no means of enforcing the desired absence for the fortnight before birth. This is always a great difficulty. It is vain for either the law or the employer to try to enforce absence before childbirth, if on the woman's work her living depends. "It is better to work and earn something than to stay at home and starve," is the argument; and as no layman can say when the confinement may be expected, the worker usually manages to go until the last possible moment.

So far we have considered only one set of mothers—the married women. But the illegitimate child is, for good or ill, as much of a national asset as the child born in wedlock, and as in 1904 one of every twenty-six of the children born in England and Wales was illegitimate, it is obvious that this also is a problem to be considered. A healthy child is a national possession whether or not he was born in wedlock, but the illegitimate mother—the phrase is convenient and not altogether inaccurate—has still less chance of sympathy and help than the poor married woman. Most of the voluntary agencies which help poor women in their confinements are connected with religious communities, and quite rightly regard the matter from the point of view of morals; but they are apt to forget that if they refuse help to the woman, the child, who is guiltless of the sins of either of his parents, will suffer from the hardships that befall his mother. If, as most people now feel, motherhood is a national duty, the rights of the child must outweigh the sins of the mother. But provision for the unmarried mother is a thing which can by no means be left to voluntary agencies.

Here, then, it is obvious that the State must step in, both to provide for the mother during her confinement, and to decide at what time before that she should stop work, and for how long after she should remain away from it. But in such a case legislation without provision is futile. The women will remain at their work until the last possible moment, and return to it as soon as they can set a foot to the ground. Some will be found out, but others, either by going to another workshop or through the connivance of foremen and forewomen, will succeed in returning to the work which means their bread far sooner than the law allows and than is desirable. Their own health will be injured—which means that sooner or later they will fall back upon the State for maintenance—and that of their children also will suffer. What then is to be done to prevent those evils? It is a problem that must be faced, although hitherto we have shirked the solution.

### The Example of France.

Perhaps this is one of the things which they manage better in France. It is eighteen years since there were started in France refuges where expectant mothers, married or single, were taken in for a time before going to the maternity hospital. Naturally few married women will go to these places, for they have their home duties to attend to, and only destitution will make them go to an institution. But for such as these and for the pitiful army of the unwed, a refuge is an inestimable boon, not only to themselves but to the children they bear. In the village of Villiers-le-Duc, near Dijon, where formerly the infant death-rate varied from 130 to 280 per 1,000 births, no child born alive between the year 1893 and 1903 succumbed within the first year of life, and there was only one still-birth. This amazing result was attained by pre-natal care. A pregnant woman, married or unmarried, has the right to demand the help of the commune if she has not sufficient means to secure proper care for herself and for her child. But to obtain this help she is compelled to make her condition known before the seventh month, when she may be visited and examined by the midwife of her choice—and in France a midwife is a woman who has had years of training—so that any special risks which could be foreseen can be noted. If after this examination the midwife thinks that the attendance of a medical man will be necessary, she must inform the authorities, and both her expenses and those of the doctor are paid out of the "free medical aid fund." To encourage the mother to give herself a fair chance of convalescence, she is paid a franc a day for six days if she remains in bed, but forfeits this donation if she gets up sooner! Has English legislation ever thought of such consideration for the risks of the puerperium?

### Infant Mortality.

With regard to the feeding of the infant we have again a lesson to learn from France. Messieurs Blin and Blin, of Elbeuf, by way of encouraging breast-feeding, promised 100 francs to each of their employées who nursed her child for twelve months. Has any English employer thought of this? The nearest we have come to it was when the Mayor of Huddersfield promised a donation of £1 for every child that completed its first year of life, and this gift has had great effect in lessening infant mortality.

In short, it seems that infant mortality, like so many other matters, is an economic problem. Women do not desire to work in a factory, or to engage in the even more trying conditions of the so-called "home-worker" until just before their children are born, if it can be avoided. Neither do they want to go back to work too soon, if they can live without it. The avoidance of suckling the child may be a more selfish matter, though mothers of the poorer classes who have tried both methods usually declare that the trouble of preparing "bottles" in even the most casual way is far greater than that of breast-feeding. But unless, as in Spain, employers are compelled to give some time for mothers to suckle their children without making deductions from their wages, economic compulsion will make them resort to artificial feeding—and that by no means of a too-careful kind. This is a matter for future consideration, and perhaps for future legislation. But meanwhile it is gratifying to know that the importance of successful motherhood has received national recognition, and that Britain is no longer content to be classed with those nations "where wealth accumulates and men decay."

